# Automated percent mammographic density, mammographic texture variation, and risk of breast cancer: a nested case-control study

**DOI:** 10.1038/s41523-021-00272-2

**Published:** 2021-05-31

**Authors:** Erica T. Warner, Megan S. Rice, Oana A. Zeleznik, Erin E. Fowler, Divya Murthy, Celine M. Vachon, Kimberly A. Bertrand, Bernard A. Rosner, John Heine, Rulla M. Tamimi

**Affiliations:** 1 Clinical and Translational Epidemiology Unit, Department of Medicine, Mongan Institute, Massachusetts General Hospital and Harvard Medical School, Boston, MA USA; 2 Channing Division of Network Medicine, Department of Medicine, Brigham and Women’s Hospital and Harvard Medical School, Boston, MA USA; 3 Division of Population Sciences, H. Lee Moffitt Cancer Center & Research Institute, Tampa, FL USA; 4 Department of Health Sciences Research, Mayo Clinic, Rochester, MN USA; 5 Slone Epidemiology Center, Boston University, Boston, MA USA; 6 Department of Epidemiology, Harvard T. H. Chan School of Public Health, Boston, MA USA; 7 Department of Population Health Sciences, Weill Cornell Medicine, New York, NY USA

**Keywords:** Epidemiology, Risk factors, Cancer epidemiology

## Abstract

Percent mammographic density (PMD) is a strong breast cancer risk factor, however, other mammographic features, such as V, the standard deviation (SD) of pixel intensity, may be associated with risk. We assessed whether PMD, automated PMD (APD), and V, yielded independent associations with breast cancer risk. We included 1900 breast cancer cases and 3921 matched controls from the Nurses’ Health Study (NHS) and the NHSII. Using digitized film mammograms, we estimated PMD using a computer-assisted thresholding technique. APD and V were determined using an automated computer algorithm. We used logistic regression to generate odds ratios (ORs) and 95% confidence intervals (CIs). Median time from mammogram to diagnosis was 4.1 years (interquartile range: 1.6–6.8 years). PMD (OR _per SD_:1.52, 95% CI: 1.42, 1.63), APD (OR _per SD_:1.32, 95% CI: 1.24, 1.41), and V (OR _per SD_:1.32, 95% CI: 1.24, 1.40) were positively associated with breast cancer risk. Associations for APD were attenuated but remained statistically significant after mutual adjustment for PMD or V. Women in the highest quartile of both APD and V (OR _vs Q1/Q1_: 2.49, 95% CI: 2.02, 3.06), or PMD and V (OR _vs Q1/Q1_: 3.57, 95% CI: 2.79, 4.58) had increased breast cancer risk. An automated method of PMD assessment is feasible and yields similar, but somewhat weaker, estimates to a manual measure. PMD, APD and V are each independently, positively associated with breast cancer risk. Women with dense breasts and greater texture variation are at the highest relative risk of breast cancer.

## Introduction

Mammographic density is one of the strongest risk factors for breast cancer, with a four- to six-fold greater breast cancer risk in women with the highest vs. lowest levels of density^1–3^. Research identifying mechanisms for these associations, or how changes in density affect risk, are limited by our reliance on visual estimation (i.e., Breast Imaging Reporting Data and Reporting System (BI-RADS)) or operator-assisted thresholding methods which require inputs by a trained user (e.g., Cumulus)^4^, that are labor intensive and prone to intra- and inter-reader variability^5^. To address this need we developed APD, an automated approach to estimate percent mammographic density (PMD). APD is moderately correlated with operator-assisted thresholding methods (*r* = 0.70), and in a Mayo Clinic case-control study yielded stronger risk estimates when comparing extreme quartiles (operator-assisted odds ratio [OR]: 3.8, 95% confidence interval [CI] 2.4–6.0 vs. automated OR: 5.2, 95% CI 3.3–8.2)^6^ may help us to better understand how dense tissue is influencing breast cancer risk.

Comparing mammograms from women with similar PMD, there may be considerable heterogeneity in the appearance of dense tissue known as texture. This information is ignored in standard measurements of PMD, yet, emerging evidence demonstrates that it is related to breast cancer risk^7,8^. In addition, several studies have demonstrated that texture features are associated with breast cancer risk, independent of PMD^7,9–13^. Using an automated image analysis system, Manduca et al. identified features within several texture classes whose association with breast cancer was of similar magnitude to PMD^10^. A texture summary measure called ‘V’ captures gray-scale variation in mammograms and was a significant predictor of breast cancer in three Mayo Clinic cohort studies^14^. Comparing extreme quartiles, V was more strongly associated with breast cancer (relative risk [RR]: 3.5, 95% CI, 1.9–6.4) than was PMD (RR: 2.2, 95% CI, 1.8–2.6). While studies have demonstrated independent associations of texture features and PMD, the interrelationship between texture, automated and manual density, with respect to breast cancer risk remains unclear.

In the current nested case-control study, we evaluated the independent and joint associations of PMD, APD, and V with breast cancer risk, using data on 1900 breast cancer cases and 3921 matched controls in the Nurses’ Health Studies. Given demonstrated positive associations between PMD and breast cancer risk, one of our primary goals was to determine whether APD performed similarly and could therefore be a more accessible and reproducible mammographic breast density measure for use in research. Another primary goal was to determine whether APD, PMD, and V were each independently associated with breast cancer risk. Finding independence between these measures would provide further information about the interrelationships between their underlying phenotypes.

## Results

### Participant characteristics

Table 1 presents participant characteristics at time of mammogram according to case/control and menopausal status. The median time from mammogram to diagnosis was 4.1 years with an interquartile range from 1.6 to 6.8 years. Participant characteristics according to image resolution and by exposure quartile are presented in Supplementary Tables 1 and 2. Cases had a mean age of 53.3 years compared to 52.6 years among controls. Cases had a higher mean PMD (37.6% vs. 32.1%), APD (18.5% vs. 17.5%), and V (0.1 vs. −0.1) as compared with controls. Similar differences were observed among pre- and postmenopausal women. Hormone receptor status among cases was predominantly ER+/PR+ (54.5%).

**Table 1:** Participant characteristics at time of mammogram for breast cancer cases and controls by menopausal status.

	All		Premenopausal		Postmenopausal	
	Cases (*N* = 1900)	Controls (*N* = 3921)	Cases (*N* = 844)	Controls (*N* = 1877)	Cases (*N* = 947)	Controls (*N* = 1800)
Mean (SD)						
Age (years)	53.3 (9.0)	52.6 (8.9)	45.8 (4.5)	46 (4.4)	60.1 (6.9)	59.6 (7.5)
BMI (kg/m^2^)	25.6 (4.9)	25.9 (5.3)	25.1 (5.0)	25.6 (5.4)	25.9 (4.7)	26.1 (5.1)
PMD	37.6 (19.8)	32.1 (19.6)	46.1 (18.4)	39.3 (19.1)	29.7 (17.9)	24.9 (17.2)
APD	18.5 (4.5)	17.5 (4.7)	19.8 (3.8)	18.7 (4.2)	17.3 (4.7)	16.2 (4.9)
V	0.1 (0.9)	−0.1 (1.0)	0.4 (0.9)	0.1 (0.9)	−0.1 (1.0)	−0.3 (0.9)
Year of mammogram	1994.9 (4.5)	1995.8 (4.8)	1996.4 (4.3)	1996.8 (4.4)	1993.8 (4.2)	1994.9 (4.9)
Year of diagnosis	1999.4 (4.4)		2001 (4.2)		1998.1 (4.2)	
*N* (%)						
Cohort						
NHS	1179 (62.1)	2163 (55.2)	256 (30.3)	546 (29.1)	836 (88.3)	1458 (81)
NHSII	721 (37.9)	1758 (44.8)	588 (69.7)	1331 (70.9)	111 (11.7)	342 (19)
HT use						
Never	1109 (58.4)	2505 (63.9)	844 (100)	1877 (100)	234 (24.7)	540 (30)
Past	574 (30.2)	949 (24.2)			510 (53.9)	832 (46.2)
Current	177 (9.3)	404 (10.3)			168 (17.7)	373 (20.7)
Unknown	40 (2.1)	63 (1.6)			35 (3.7)	55 (3.1)
ER status						
ER+	1253 (65.9)		565 (66.9)		616 (65)	
ER−	279 (14.7)		135 (16)		131 (13.8)	
Unknown	368 (19.4)		144 (17.1)		200 (21.1)	
PR status						
PR+	1070 (56.3)		513 (60.8)		496 (52.4)	
PR−	429 (22.6)		178 (21.1)		228 (24.1)	
Unknown	401 (21.1)		153 (18.1)		223 (23.5)	
ER/PR status						
ER+/PR+	1036 (54.5)		500 (59.2)		476 (50.3)	
ER+/PR−	188 (9.9)		56 (6.6)		120 (12.7)	
ER−/PR−	241 (12.7)		122 (14.5)		108 (11.4)	
Unknown	435 (22.9)		166 (19.7)		243 (25.7)	

*BMI* body mass index, *PMD* percent mammographic density, *V* variation measure, *HT* Postmenopausal hormone therapy, *ER* estrogen receptor, *PR* progesterone receptor.

### PMD, APD, V, and breast cancer risk by menopausal status

PMD, APD, and V were moderately correlated (Supplementary Fig. 3) and each was positively associated with breast cancer risk (Table 2). V was more strongly correlated to APD than PMD (*r* = 0.83 vs. *r* = 0.61). Compared to the lowest quartile (Q1), individuals in the highest quartile (Q4) of PMD were almost three times more likely to develop breast cancer (OR: 2.90, 95% CI: 2.39, 3.52; *p* trend < 0.01). PMD associations were stronger among premenopausal (Q4 vs. Q1 OR: 3.56, 95% CI: 2.49, 5.08; *p* trend < 0.01) compared to postmenopausal women (Q4 vs. Q1 OR: 2.32, 95% CI: 1.78, 3.04; *p* trend < 0.01). With adjustment for V, the association was somewhat attenuated, but remained significant overall (Q4 vs. Q1 OR: 2.36, 95% CI: 1.90, 2.94; *p* trend < 0.01), and among premenopausal (Q1 vs. Q4 OR: 2.86, 95% CI: 1.94, 4.21; *p* trend < 0.01) and postmenopausal women (Q4 vs. Q1 OR 1.91, 95% CI: 1.41, 2.59; *p* trend < 0.01). For APD, compared to Q1, women in Q4 were twice as likely to develop breast cancer (overall: OR: 2.10, 95% CI: 1.75, 2.51; *p* trend < 0.01; premenopausal: OR: 2.27, 95% CI: 1.67, 3.08; *p* trend < 0.01; postmenopausal OR: 2.04, 95% CI: 1.59, 2.62; *p* trend < 0.01). When adjusted for V, comparing Q4 vs. Q1 was associated with a 36% increased breast cancer risk overall (OR: 1.36, 95% CI: 1.04, 1.77; *p* trend = 0.05), 55% among premenopausal (OR: 1.55, 95% CI: 1.03, 2.32; *p* trend = 0.05), and 25% among postmenopausal women (OR: 1.25, 95% CI: 0.85, 1.84; *p* trend = 0.53). Lastly, for V, compared to women in Q1, those in Q4 were more than twice as likely to develop breast cancer (overall OR: 2.16, 95% CI: 1.81, 2.58; *p* trend < 0.01; premenopausal OR: 2.46, 95% CI: 1.83, 3.31; *p* trend < 0.01; postmenopausal OR: 2.09, 95% CI: 1.64, 2.67; *p* trend < 0.01). Adjustment for PMD or APD attenuated the associations, but V remained associated with breast cancer risk. Associations did not differ by tumor hormone receptor status (Supplementary Table 3) and were strongest among high-resolution images (Supplementary Table 4).

**Table 2:** ORs and 95% CIs for the associations of PMD, APD, V, with breast cancer risk by menopausal status in the NHS/NHSII.

	All			Pre-menopausal			Post-menopausal		
*PMD*	*Model 1*^*a*^	*Model 1* + *V*		*Model 1*^*a*^	*Model 1* + *V*		*Model 1*^*a*^	*Model 1* + *V*	
Quartile 1 < 16	Ref	Ref		Ref	Ref		Ref	Ref	
Quartile 2 16–<30	1.47 (1.23, 1.75)	1.33 (1.10, 1.60)		1.70 (1.18, 2.46)	1.54 (1.06, 2.24)		1.37 (1.11, 1.70)	1.24 (0.98, 1.56)	
Quartile 3 30–<46	1.97 (1.64, 2.37)	1.64 (1.33, 2.01)		2.20 (1.54, 3.13)	1.79 (1.22, 2.63)		1.94 (1.53, 2.46)	1.63 (1.24, 2.14)	
Quartile 4 ≥ 46	2.90 (2.39, 3.52)	2.36 (1.90, 2.94)		3.56 (2.49, 5.08)	2.86 (1.94, 4.21)		2.32 (1.78, 3.04)	1.91 (1.41, 2.59)	
*p* trend	<0.01	<0.01		<0.01	<0.01		<0.01	<0.01	
Per 1 SD	1.52 (1.42, 1.63)	1.43 (1.33, 1.54)		1.5 3 (1.39, 1.70)	1.45 (1.31, 1.62)		1.47 (1.33, 1.63)	1.37 (1.21, 1.54)	
*APD*	*Model 1*^*a*^	*Model 1* + *V*		*Model 1*^*a*^	*Model 1* + *V*		*Model 1*	*Model 1* + *V*	
Quartile 1 < 14	Ref	Ref		Ref	Ref		Ref	Ref	
Quartile 2 14–<18	1.52 (1.28, 1.80)	1.25 (1.04, 1.52)		1.61 (1.17, 2.20)	1.36 (0.97, 1.90)		1.49 (1.20, 1.85)	1.20 (0.94, 1.55)	
Quartile 3 18–<21	1.56 (1.31, 1.87)	1.13 (0.89, 1.42)		1.95 (1.43, 2.66)	1.46 (1.01, 2.11)		1.27 (1.00, 1.62)	0.88 (0.63, 1.22)	
Quartile 4 ≥ 21	2.10 (1.75, 2.51)	1.36 (1.04, 1.77)		2.27 (1.67, 3.08)	1.55 (1.03, 2.32)		2.04 (1.59, 2.62)	1.25 (0.85, 1.84)	
*p* trend	<0.01	0.05		<0.01	0.05		<0.01	0.53	
Per 1 SD	1.32 (1.24, 1.41)	1.15 (1.04, 1.28)		1.37 (1.23, 1.53)	1.21 (1.03, 1.41)		1.28 (1.17, 1.39)	1.10 (0.94, 1.29)	
*V*	*Model 1*^*a*^	*Model 1* + *PMD*	*Model 1* + *APD*	*Model 1*^*a*^	*Model 1* + *PMD*	*Model 1* + *APD*	*Model 1*^*a*^	*Model 1* + *PMD*	*Model 1* + *APD*
Quartile 1<−0.76	Ref	Ref	Ref	Ref	Ref	Ref	Ref	Ref	Ref
Quartile 2−0.76–<−0.09	1.43 (1.21, 1.70)	1.26 (1.05, 1.50)	1.23 (1.00, 1.51)	1.85 (1.36, 2.52)	1.60 (1.17, 2.18)	1.55 (1.10, 2.19)	1.22 (0.98, 1.53)	1.11 (0.88, 1.39)	1.11 (0.85, 1.46)
Quartile 3 −0.09–0.56	1.69 (1.42, 2.01)	1.31 (1.09, 1.58)	1.32 (1.03, 1.69)	1.98 (1.47, 2.68)	1.53 (1.12, 2.09)	1.49 (1.00, 2.21)	1.57 (1.24, 1.99)	1.28 (0.99, 1.64)	1.35 (0.95, 1.90)
Quartile 4 > 0.56	2.16 (1.81, 2.58)	1.52 (1.26, 1.84)	1.60 (1.21, 2.11)	2.46 (1.83, 3.31)	1.79 (1.32, 2.45)	1.73 (1.13, 2.67)	2.09 (1.64, 2.67)	1.51 (1.14, 1.98)	1.72 (1.15, 2.58)
*p* trend	<0.01	<0.01	<0.01	<0.01	<0.01	0.05	<0.01	<0.01	<0.01
Per 1 SD	1.32 (1.24, 1.40)	1.15 (1.08, 1.23)	1.18 (1.07, 1.31)	1.32 (1.20, 1.45)	1.18 (1.07, 1.31)	1.17 (1.01, 1.34)	1.30 (1.19, 1.42)	1.14 (1.03, 1.26)	1.20 (1.02, 1.40)

*OR* odds ratio, *CI* confidence interval, *PMD* percent mammographic density, *APD* automated percent density, *V* variation measure, *NHS* Nurses’ Health Study, *NHSII* Nurses’ Health Study II.

^a^Model 1 is adjusted for: Age (continuous), fasting status, time of blood draw, body mass index (kg/m2), menopausal status (premenopausal, postmenopausal, unknown), hormone therapy use (never, past, current, unknown), mammography read batch (batch 1, batch 2, batch 3).

### PMD, APD cross-classified with V and breast cancer risk

V was positively associated with breast cancer risk within each quartile of either PMD or APD (Table 3). Women in Q4 of PMD and V had more than three times higher risk of breast cancer compared to women in Q1 of PMD and V (OR: 3.57; 95% CI: 2.79, 4.58; *p* interaction = 0.75). Women in Q4 of PMD, but Q1 of V had more than twice the risk of breast cancer compared to those who were low on both measures (OR: 2.50, 95% CI: 1.51, 4.14). High V (Q4) coupled with low PMD (Q1) was not associated with increased risk of breast cancer (OR: 1.40, 95% CI: 0.48, 4.07). Similarly, women in the highest APD and V quartiles, had two and a half times greater risk of breast cancer compared to those with the lowest APD and V (OR: 2.49, 95% CI: 2.02, 3.06; *p* heterogeneity = 0.75). The patterns were similar, though the magnitude of association was stronger when analyses were restricted to high-resolution images only (Supplementary Table 5).

**Table 3:** ORs and 95% CIs for the associations between breast cancer risk and APD and PMD, cross-classified with V, in NHS/NHSII^a^.

	V				
PMD	Quartile 1 < −0.76	Quartile 2 −0.76–< −0.09	Quartile 3 −0.09–0.56	Quartile 4 > 0.56	*p* interaction
Quartile 1 < 16	Ref	1.28 (0.96, 1.69)	1.32 (0.86, 2.04)	1.40 (0.48, 4.07)	0.75
Quartile 2 16–<30	1.35 (1.00, 1.83)	1.71 (1.31, 2.22)	1.83 (1.38, 2.43)	1.67 (1.17, 2.38)	
Quartile 3 30–<46	1.43 (0.89, 2.28)	2.01 (1.47, 2.73)	2.01 (1.53, 2.64)	2.73 (2.11, 3.52)	
Quartile 4 ≥ 46	2.50 (1.51, 4.14)	2.94 (2.12, 4.09)	3.22 (2.45, 4.24)	3.57 (2.79, 4.58)	
*APD*					*p* interaction
Quartile 1 < 14	Ref	1.50 (1.10, 3.04)	1.81 (0.88, 3.73)	2.81 (0.62, 12.7)	0.75
Quartile 2 14–<18	1.52 (1.12, 2.07)	1.56 (1.26, 1.93)	2.01 (1.54, 2.64)	2.10 (1.38, 3.21)	
Quartile 3 18–<21	0.56 (0.13, 2.50)	1.52 (1.15, 2.02)	1.61 (1.27, 2.03)	2.19 (1.70, 2.80)	
Quartile 4 ≥ 21	Not estimated	2.02 (1.25, 3.26)	2.09 (1.62, 2.69)	2.49 (2.02, 3.06)	

*OR* odds ratio, *CI* confidence interval, *PMD* percent mammographic density, *APD* automated percent density, *V* variation measure, *NHS* Nurses’ Health Study, *NHSII* Nurses’ Health Study II.

^a^Models are adjusted for: Age (continuous), fasting status, time of blood draw, body mass index (kg/m2), menopausal status (premenopausal, postmenopausal, unknown), hormone therapy use (never, past, current, unknown), mammography read batch (batch 1, batch 2, batch 3).

### Independent associations of PMD and APD with breast cancer risk

PMD and APD measures were independently associated with breast cancer risk (Table 4). Without adjustment for APD, each SD increase in PMD was associated with a 52% increased breast cancer risk (OR: 1.52, 95% CI: 1.42, 1.63). With adjustment for APD, the association was attenuated but remained significant (OR: 1.43, 95% CI: 1.33, 1.55). Similarly, without adjustment for PMD, APD was associated with a 33% increased breast cancer risk per SD. After adjustment for PMD, there was a 14% increase in breast cancer risk per SD (OR: 1.14, 95% CI: 1.06, 1.22).

**Table 4:** ORs and 95% CIs for the associations between breast cancer risk and APD and PMD with mutual adjustment, in NHS/NHSII^a^.

PMD	Not adjusted for APD	Adjusted for APD
Quartile 1 < 16	Ref	Ref
Quartile 2 16–<30	1.47 (1.23, 1.75)	1.33 (1.11, 1.61)
Quartile 3 30–<46	1.97 (1.64, 2.37)	1.66 (1.35, 2.05)
Quartile 4 ≥ 46	2.90 (2.39, 3.52)	2.40 (1.91, 3.00)
*p* trend	<0.0001	<0.0001
Per 1 SD	1.52 (1.42, 1.63)	1.43 (1.33, 1.55)

*APD*	Not adjusted for PMD	Adjusted for PMD
Quartile 1 < 14	Ref	Ref
Quartile 2 14–<18	1.52 (1.28, 1.80)	1.32 (1.11, 1.57)
Quartile 3 18–<21	1.57 (1.31, 1.87)	1.15 (0.96, 1.40)
Quartile 4 ≥ 21	2.10 (1.75, 2.51)	1.40 (1.15, 1.70)
*p* trend	<0.0001	0.0048
Per 1 SD	1.33 (1.24, 1.41)	1.14 (1.06, 1.22)

*OR* odds ratio, *CI* confidence interval, *PMD* percent mammographic density, *APD* automated percent density, *V* variation measure, *NHS* Nurses’ Health Study, *NHSII* Nurses’ Health Study II.

^a^Models adjusted for: Age (continuous), fasting status, time of blood draw, body mass index (kg/m2), menopausal status (premenopausal, postmenopausal, unknown), hormone therapy use (never, past, current, unknown), mammography read batch (batch 1, batch 2, batch 3). PMD adjusted for continuous automated percent MD and vice versa.

## Discussion

In this large, nested case-control study, we investigated the associations of PMD, APD, and V, a summary measure of mammographic greyscale variation, with breast cancer risk among 1900 breast cancer cases and 3921 controls. This study adds to the literature by simultaneously evaluating two density breast measures (PMD and APD) and a texture measure V. We demonstrated that PMD, APD, and V were independently associated with breast cancer risk. However, PMD was more strongly associated with breast cancer risk than were APD or V. When PMD and APD were modeled together, the association of APD with breast cancer risk was more attenuated than PMD. Associations were generally stronger among premenopausal women and high-resolution images but did not vary by hormone receptor status.

We observed independent and joint effects of V, APD, and PMD on breast cancer risk demonstrating that both the relative amount of fibroglandular tissue and its greyscale variation contribute to breast cancer risk. While all three measures were associated with breast cancer risk, PMD had the strongest magnitude of association. This differs from the Mayo Clinic Studies, where V yielded higher risk estimates than PMD in two of three included cohorts, and in one they were equivalent^6,14^. In that study the authors conclude that V and PMD are at least equivalent and our findings are consistent with an interpretation that these factors are at least equivalent in their association with breast cancer risk. Further study is required to determine their precise interrelationship. As reviewed in Gastounioti et al. (2016) parenchymal texture classifiers have been assessed with respect to breast cancer risk in at least 20 studies^8^. A prospective cohort study by Wanders et al. (2018) found that percent dense volume and texture, as assessed using an algorithm the authors previously developed^15^, were each associated with breast cancer risk^12^. While risk prediction was not the goal of our study, Winkel et al. (2016) found that combining measures of PMD (BI-RADS), parenchymal patterns (Tabar’s classification), and an automated texture measure, improved breast cancer risk prediction. The area under the curve (AUC) for either measure alone ranged from 0.63 to 0.65, while inclusion of all three yielded an AUC of 0.69^16^. Importantly, while these features may ultimately improve breast cancer risk prediction, they can also yield insights into breast cancer etiology by identifying specific breast structures implicated in cancer development.

Despite the somewhat stronger associations observed for PMD, validation of APD has important implications for future research^17,18^. Manual measures are subject to reader differences^19^, measurement error^20^, and are time-intensive. An automated measure, such as APD, can provide a more reliable measure, suitable for use in risk assessment, mandatory breast density notification, and measuring changes in density. Inconsistent breast density measurement could bias study results toward the null^21^, lead to unreliable risk stratification if included in risk assessment models, or misinformed decision-making regarding screening intervals and modalities after legally mandated breast density notification^22,23^, and reduce reliability when assessing change in PMD^24^. We have demonstrated that including breast density in a breast cancer risk assessment model improves discriminatory accuracy^25,26^ and a recent paper shows that the Tyrer–Cuzick model with density can provide useful data at on risk for at least 10 years^27^. However, those estimates were based on a single baseline breast density assessment. Cuzick et al^24^. noted that the reliability for change in BI-RADS density was only moderate (*r* = 0.48–0.67) when evaluating mammograms 10 years apart. As changes in PMD over time are likely to be small^28^, a continuous measure, more sensitive than BI-RADS which has just four categories, is needed. In recent years, several automated measurement tools have been developed to assess volumetric or area-based breast density^18,29–32^. These new measurement approaches are essential for PMD use in breast cancer risk assessment and screening decision-making. Yet, our finding that PMD yielded stronger associations with breast cancer risk than did APD or V, two automated measures, suggests that there is information captured through operator-assisted thresholding that is not captured through automation. Continued effort is needed to compare the strengths and weaknesses of these approaches and develop standardized methods for clinical PMD assessment.

Study strengths include the use of prospective data from the Nurses Health Studies, two cohorts with validated disease ascertainment, and comprehensive data on breast cancer risk factors, tumor hormone receptor status, measures of PMD, APD, and texture features. This study has several important limitations. Our study focused on V, a summary texture measure, but there are many other features that have potential implications. For example, Malkov et al. (2016) examined 46 breast texture features and identified 15 that were significantly associated with breast cancer risk (*p* < 0.05), several of which were only weakly correlated with PMD^9^. In future studies, we will assess the relative importance of multiple features, independently and in combination with PMD. There is potential measurement error in the exposure assessment, particularly given that mammograms in this study were collected from across the United States across many years and images had multiple image resolution levels. To address this, we conducted sensitivity analyses and found the strongest associations among high-resolution images. This study utilized digitized film mammograms. As of November 1, 2019, 99.9% (21,156/21,182) of all accredited mammography units in the US are digital^33^. However, Nielsen et al. found that while differences in population characteristics and imaging technology did affect texture feature measurement, these factors did not impact the association between texture and breast cancer risk^7^. Further, Vachon et al. showed that associations with breast cancer were similar between full field digital mammogram image types (raw or processed) and digitized film^34^, demonstrating that these measures are stable phenotypes across image acquisition approaches and our findings are valid despite the use of digitized film mammograms. The relative consistency of our results compared to other studies with different imaging modalities confirms this assertion. Lastly, the Nurses’ Health Studies consist of predominantly white women. Future studies should assess associations among more diverse populations.

In conclusion, we demonstrate that APD is feasible and yields risk estimates that are similar to, but somewhat weaker than, a more labor-intensive and less reproducible manual measure. Our finding of independent and joint associations of PMD, APD, and V provides insights into breast cancer etiology. This study supports existing evidence that the amount dense tissue and its heterogeneity are both important factors in breast cancer risk.

## Methods

### Study population

Our study population includes 1900 breast cancer cases and 3921 matched controls from the Nurses’ Health Study (NHS) and Nurses’ Health Study II (NHSII). NHS began in 1976 with 121,700 female registered nurses aged 30–55 from 11 states. NHSII began in 1989 with 116,429 female registered nurses, aged 25–42, from 14 states. Participants in each cohort completed a baseline questionnaire at enrollment and are followed by biennially mailed questionnaires to collect information on newly diagnosed diseases, exposures, and covariates.

### Mammogram collection and processing

Mammogram collection was conducted within the NHS and NHSII breast cancer case-control studies nested in the blood subcohorts^35^. One or two controls were matched to breast cancer cases on age, menopausal status at blood draw and diagnosis, current hormone therapy use, month, time of day, fasting status at time of blood collection, and luteal day (NHSII timed samples only). We collected pre-diagnostic screening mammograms conducted as close as possible to the blood draw date, but before June 1, 2004 (NHS) or June 1, 2007 (NHSII). We collected additional mammograms conducted around 1997 from NHSII cases and controls who participated in the NHSII cheek cell collection. In total, mammograms were collected from 2062 breast cancer cases and 4196 matched controls. We excluded 162 cases and 275 controls due to missing data on V or BMI. Film mammogram cranio-caudal (CC) views of each breast were digitized with a Lumysis 85 laser film scanner or a VIDAR CAD PRO Advantage scanner (VIDAR Systems Corporation, Herndon, VA, USA). The correlation between percent density measures from the two scanners was 0.88^36^. Cohort participants provided written informed consent. The study protocol was approved by the institutional review boards of the Brigham and Women’s Hospital and Harvard T.H. Chan School of Public Health, and those of participating registries as required.

### Manual percent mammographic density (PMD)

PMD was assessed using Cumulus, a computer-assisted thresholding software program (University of Toronto, Toronto, Canada), from digitized film mammograms (craniocaudal view)^37^. PMD was calculated as dense breast area divided by the total breast area. All images were assessed by a single reader (within-person intra-class correlation coefficient > 0.90)^38^. Observed inter-batch variability was accounted for using methods described elsewhere^39,40^. PMD measures in the left and right breast were averaged.

### Automated percent mammographic density (APD)

This APD method detects small dense regions on a mammogram after applying a wavelet high-pass filter and produces an output analogous to that of the operator-assisted PMD. The basic algorithm and its validation were described previously and details are provided in Supplementary Methods^6,41,42^. To avoid the chest wall, detection is performed in cranioclaudial views only. First, the breast area was detected creating a binary mask shown in Supplementary Fig. 1 using a method described in related work. Because the images in this study had high variability and contained many artifacts in the non-breast area, automated segmentation for each image was evaluated visually. When the breast area segmentation was deemed not appropriate for further processing, manual intervention was applied. The breast area detection performance is provided in the next section. After this step, modifications to the APD algorithm were required to account for the relatively low resolution of the mammograms used in this study (171, 232, or 300 µm) described in detail in the next section; the main modification is based on multiplying a given mammogram with a noise field producing an image illustrated in Supplementary Fig. 2 and then filtering this image in place of the raw image.

### Automated percent mammographic density (APD) technique and modifications

The density detection is based on the signal dependent noise characteristic in mammograms, analyzed in the high-pass wavelet filtered image. Due to the low resolution, the noise characteristic was not strong enough to characterize the density in the filtered images, requiring algorithm modifications. These modifications are described here within the context of the algorithm flow described in the main report. In the unmodified algorithm, the digitized images were first transformed (a pixel mapping) described previously^6^ [and then processed with a high-pass wavelet filter. The density detection is performed in two stages in the wavelet filtered image, differing in thresholds based on predefined significance levels (i.e., operating parameters). To boost the noise signal, each transformed image was multiplied with a different realization of a zero mean unit variance Gaussian noise field and then scaled to 14 bit (integer) prior to applying the wavelet high-pass filter. Examples of this process are shown in Supplementary Fig. 2. The automated density detection was then performed by replacing a given transformed image with its corresponding noise field exemplified in Supplementary Fig. 2. These modified images were then filtered and the density detection was constrained to the breast area using the algorithm described previously^6,41,42^. In the first stage, a reference variance (i.e., the global adipose variance) is estimated using the entire breast area in the filtered image. A small search window (4 × 4 pixels) is scanned across the filtered image. At each window location, a chi-square statistical test is performed by comparing the local variance calculated within the window with the reference variance to decide if the region is dense or not as represented in the *raw* image (i.e., larger local variation is more likely to correspond to a region with high breast density in the *raw* image). The connection between the local variation in the filtered image and the digitized (raw) image density characteristics was demonstrated previously^41,43^. This detection method is applied a second time in the filtered image by updating (refining) the reference variance. The reference variance is updated by calculating the variance across the breast area in the filtered image using regions not labeled as dense in the first detection stage. Because of the noise field multiplication modification, we used 300 images (100 images from each resolution) as a test set to adjust the algorithm’s parameters. Each training image had the corresponding density determined with Cumulus. The operating points were determined by finding the parameters that gave the optimal correlation with the Cumulus measures. These correspond to significance levels used in the first and second detection stages discussed above respectively: 0.001 and 0.0001. The entire dataset was then processed.

### Breast area segmentation performance

This algorithm produced acceptable breast area detection in most samples. However, there were both total and partial failures that were corrected manually. In some instances, artifacts external to the breast area remained that induced failures in subsequent segmentation processing steps; these failures occurred ~3% of the images, which were corrected manually. Approximately 6% on the images had a digitizing process anomaly (i.e., the film was not position correctly when fed into the digitizer) noted as bright wedges on the vertical borders of the images. After the breast area detection, these over contrasted areas were removed manually.

### Automated summary measure of texture features (‘V’)

V is an automated measure that captures gray-scale variation on a mammogram. The algorithm was described previously and is briefly outlined here^14,42^. The breast is first segmented from the background. The breast area is then eroded by 25% along a radial direction to eliminate the region corresponding to where the breast was not in contact with the compression paddle (an approximation) during the image acquisition^44^. The erosion process is illustrated in Supplementary Fig. 1. This erosion step reduces unwanted variation in the V calculation. V is calculated as the standard deviation (SD) of the pixel values within the eroded breast area. This is a continuous measure that is not synthetically normalized. There are no operating parameters or thresholds required for this measure (i.e., training data is not required), although the background segmentation processing required preliminary analyses. Because the mammograms were at various resolutions, they were resolution-normalized prior to generating V. The V distributions were also normalized to account for intensity scale differences across the various forms of digitized mammograms. Extensive additional processing steps were required and are described in related work^45^. Example images with high and low levels of PMD and V are shown in Supplementary Fig. 2.

### Statistical analyses

We used unconditional logistic regression to determine the association between PMD measures, V, and breast cancer risk, while adjusting for matching factors and the following potential confounders: age at mammogram (years), body mass index (kg/m^2^), menopausal status (premenopausal, postmenopausal, unknown), hormone therapy use (never, past, current, unknown), mammogram read batch (batch 1, batch 2, batch 3). APD, PMD, and V were categorized into quartiles based on their distribution in controls. We tested for linear trend using category medians as a continuous variable. We also evaluated each measure as a continuous variable and present effect estimates for a one SD increase to account for scale differences. To determine whether each measure was independently associated with breast cancer risk, we present models for PMD or APD adjusted for V, V adjusted for PMD or APD, and APD adjusted for PMD and vice versa. To assess potential interaction, we used likelihood ratio tests with nine degrees of freedom to compare a model with cross-classified quartiles of PMD (or APD) and V to a model with PMD (or APD) and V in quartiles. We conducted secondary analyses: (1) by estrogen (ER) and progesterone (PR) receptor status (ER+/PR+, ER+/PR−, or ER−/PR−) and (2) restricted to high-resolution images. All analyses use *α* = 0.05 for statistical significance. All statistical tests were two sided. All analyses were performed using SAS software (version 9.4 SAS Institute, Cary, NC, USA).

### Reporting summary

Further information on research design is available in the Nature Research Reporting Summary linked to this article.

## Supplementary information

Supplementary Information

Reporting Summary

## Data Availability

The data generated and analyzed during this study are described in the following data record: 10.6084/m9.figshare.14511756^46^. The data that support the findings of this study are available from the Nurses’ Health Studies, however they are not publicly available. Investigators interested in using the data can request access, and feasibility will be discussed at an investigators’ meeting. Limits are not placed on scientific questions or methods, and there is no requirement for co-authorship. Additional data sharing information and policy details can be accessed at http://www.nurseshealthstudy.org/researchers.

## Code availability

Algorithms used to generate automated percent density and V are available upon request to the corresponding author.
