# Supplementary material for: Automated percent mammographic density, mammographic texture variation, and risk of breast cancer: a nested case-control study
Source: NPJ Breast Cancer. 2021 May 31;7:68. doi: 10.1038/s41523-021-00272-2 (PMC8166859; doi:10.1038/s41523-021-00272-2)
Supplement: Supplementary file 1 — Supplementary Information [file 41523_2021_272_MOESM1_ESM.pdf]

Supplementary Materials

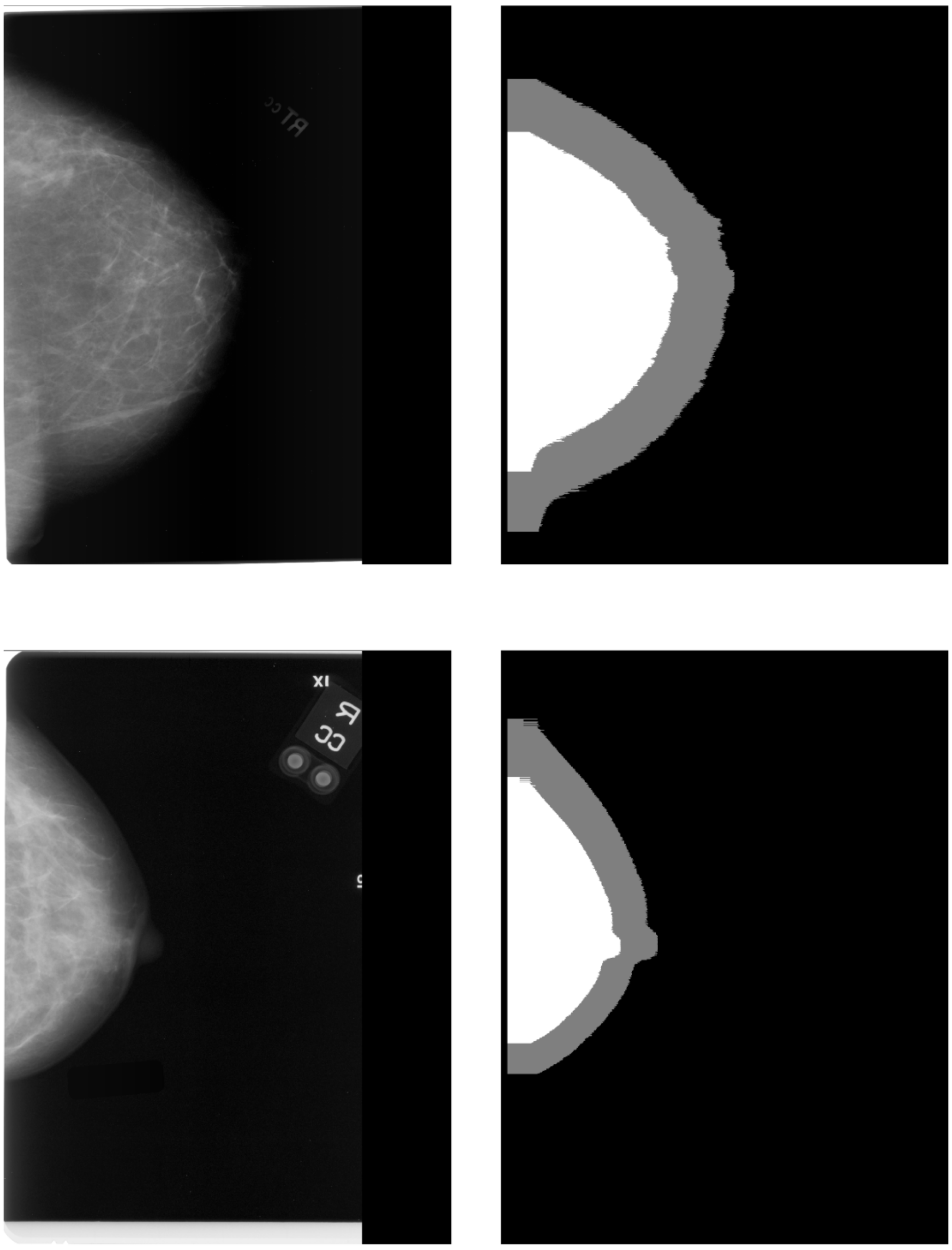

Supplementary Figure 1. Segmentation and erosion examples: The figures on the left show typical mammograms used for this study. The images on the right show the corresponding breast area segmentations and the eroded breast areas. The white area in the segmented images (right images) shows the 75% erosion.

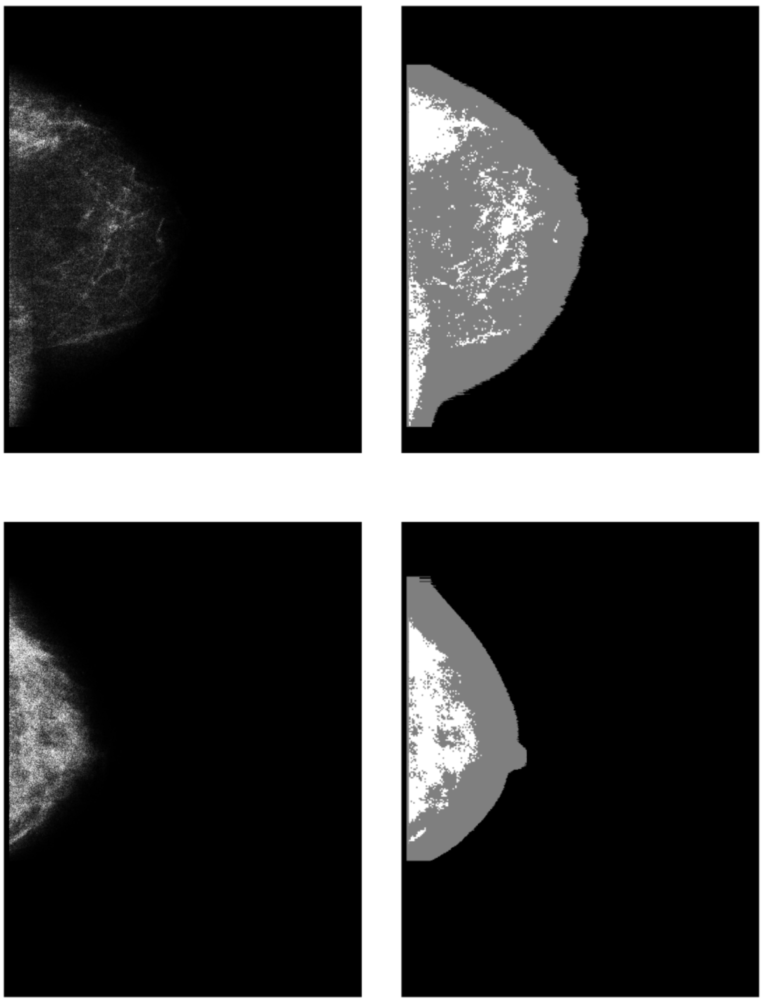

Supplementary Figure 2. Modified Breast Density Detection: Figures on the left (top and bottom) show the modified digitized (*raw)* images after the noise field multiplication. These images have been over contrasted for illustration purposes. The images on the right show the respective density detection outputs.

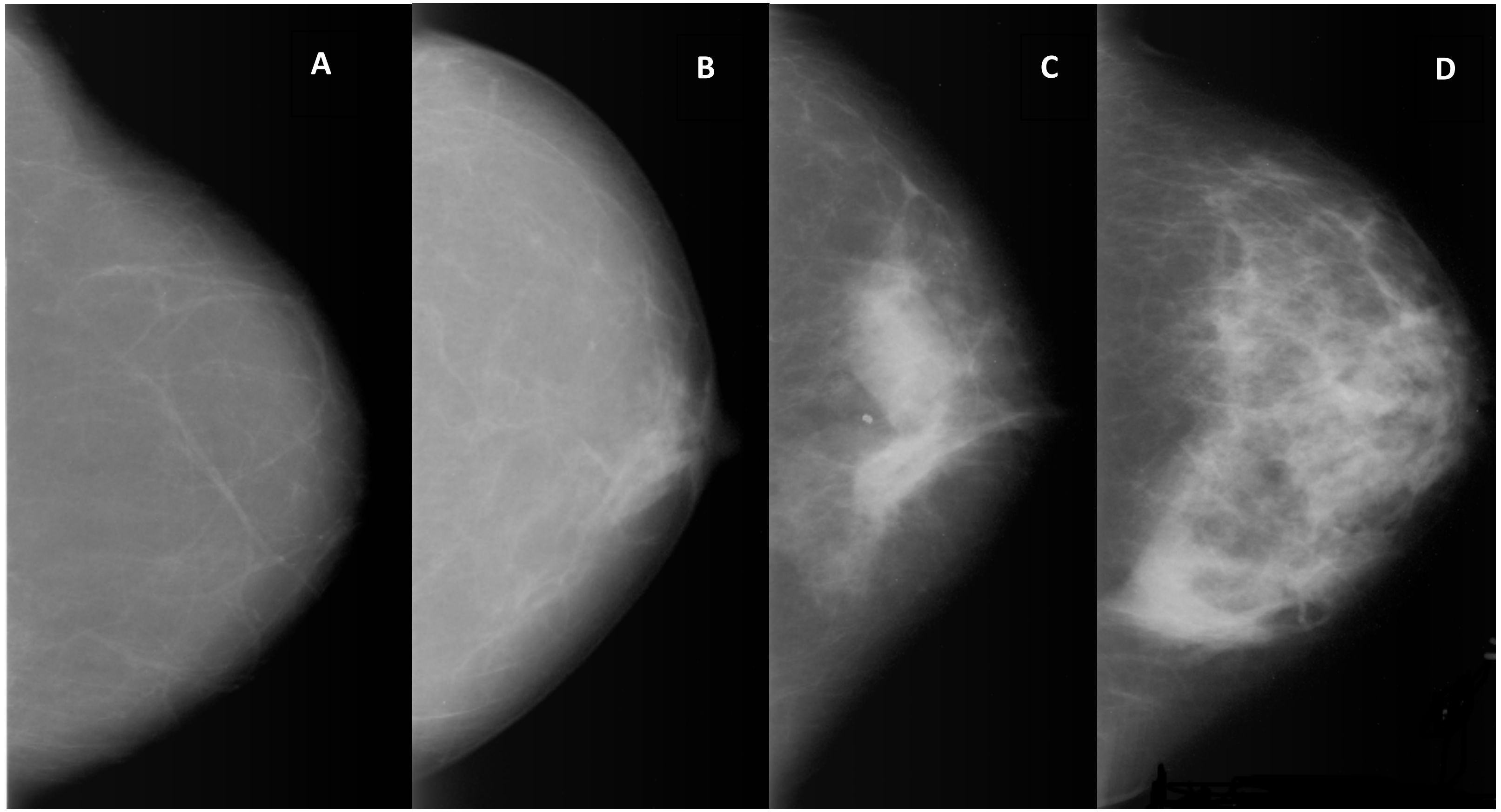

Supplementary Figure 3. Example mammograms with high or low levels of manual percent mammographic density (PMD) and V. Mammograms are shown with A) low PMD and low V (PMD = 1.3%, V = -2.8,); and B) high PMD and low V (PMD=69.0%, V = -1.9), C) low PMD and high V (PMD=15.9%, V = 1.2), D) high PMD and high V PMD=71.1%, V = 2.1).

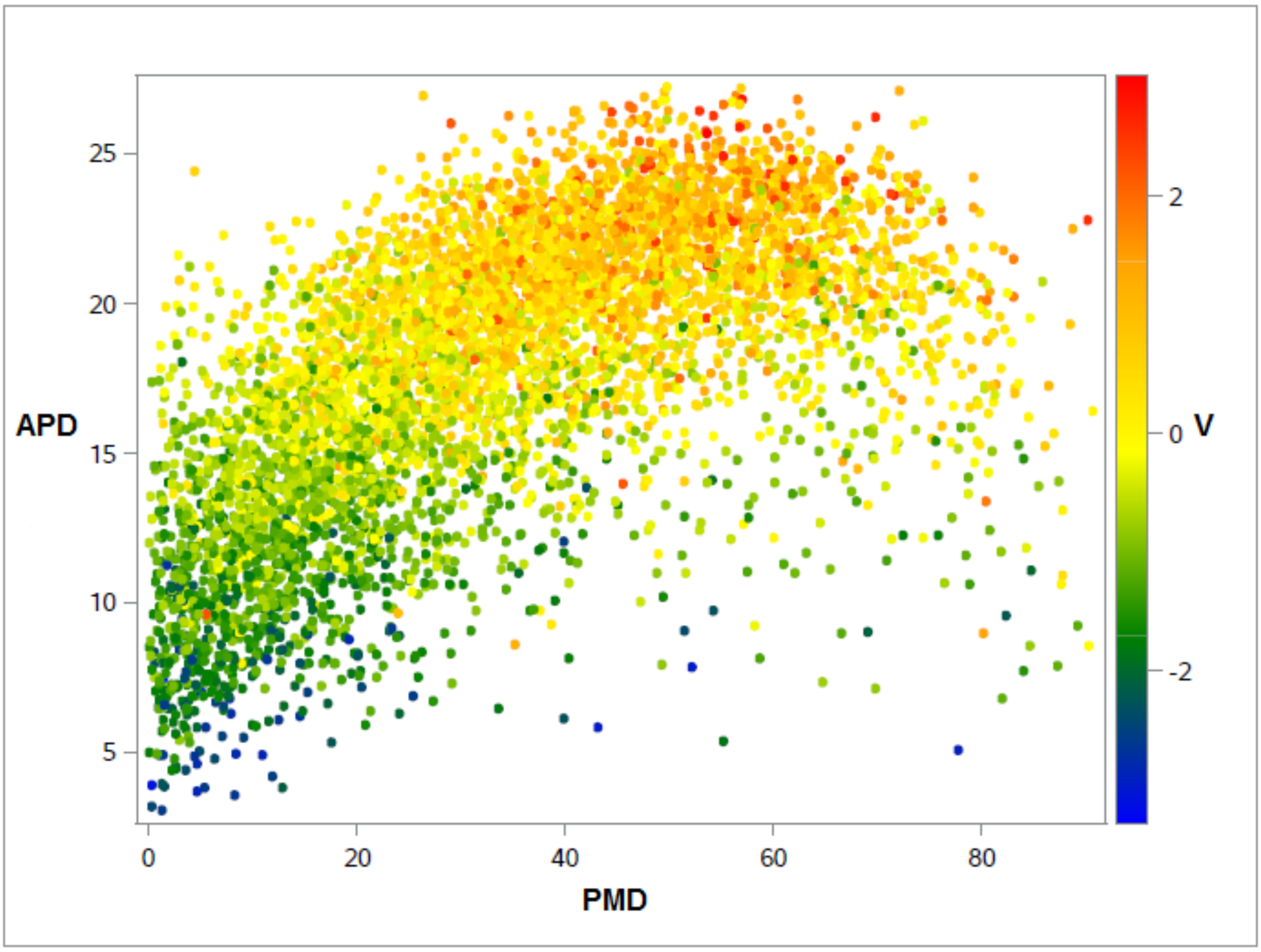

	Spearman correlation coefficients		
	**PMD**	**APD**	**V**
**PMD**	1.00	0.67	0.61
**APD**	0.67	1.00	0.83
**V**	0.61	0.83	1.00

Supplementary Figure 4. Distribution of automated percent mammographic density (APD) by manual percent density (PMD) and V in in the NHS/NHSII. APD and PMD are shown on the x- and y- axis respectively. V level is shown by dot color with low scores in bright blue, mid-range scores in yellow, and the highest scores in red. The table displays spearman correlation coefficients for each metric.

Supplementary Table 1. Participant characteristics at time of mammogram for breast cancer cases and controls by image resolution, in the NHS/NHSII

	High resolution images		Medium resolution images		Low resolution images	
	Cases (N = 990)	Controls (N = 2207)	Cases (N = 197)	Controls (N = 431)	Cases (N = 713)	Controls (N = 1283)
Mean (SD)						
Age (years)	49.8(8.6)	49.7(8.4)	55.9(9.8)	54.1(9.1)	57.4(7.2)	57.1(7.6)
BMI (kg/m2)	24.9(4.5)	25.1(4.8)	30(5.7)	31.2(6.6)	25.3(4.5)	25.5(4.5)
PMD	41.4(17.8)	36.2(17.9)	26.1(16.1)	22.7(16.4)	35.4(21.8)	28.3(21.3)
APD	19.7(3.7)	18.7(4.2)	16.1(4.1)	14.8(4.6)	17.4(5)	16.4(5)
Variation measure (V)	0.2(0.9)	0(0.9)	-0.3(0.9)	-0.5(1)	0.1(1)	-0.1(1)
Year of mammogram	1997.2(4.0)	1997.9(4.0)	1996.9(3.9)	1997.9(4.7)	1991.1(1.9)	1991.4(2.6)
N (%)						
Cohort						
NHS	345(34.8)	670(30.4)	124(62.9)	218(50.6)	710(99.6)	1275(99.4)
NHSII	645(65.2)	1537(69.6)	73(37.1)	213(49.4)	3(0.4)	8(0.6)
Menopausal status						
Premenopausal	605(61.1)	1342(60.8)	70(35.5)	172(39.9)	169(23.7)	363(28.3)
Postmenopausal	342(34.5)	761(34.5)	115(58.4)	223(51.7)	490(68.7)	816(63.6)
Unknown	43(4.3)	104(4.7)	12(6.1)	36(8.4)	54(7.6)	104(8.1)
HT use						
Never	682(68.9)	1547(70.1)	106(53.8)	246(57.1)	321(45)	712(55.5)
Past	249(25.2)	497(22.5)	63(32)	113(26.2)	262(36.7)	339(26.4)
Current	47(4.7)	144(6.5)	24(12.2)	67(15.5)	106(14.9)	193(15)
Unknown	12(1.2)	19(0.9)	4(2)	5(1.2)	24(3.4)	39(3)
ER status						
ER+	685(69.2)		140(71.1)		428(60)	
ER-	133(13.4)		22(11.2)		124(17.4)	
Unknown	172(17.4)		35(17.8)		161(22.6)	
PR status						
PR+	582(58.8)		121(61.4)		367(51.5)	
PR-	225(22.7)		37(18.8)		167(23.4)	
Unknown	183(18.5)		39(19.8)		179(25.1)	
ER/PR status						
ER+/PR+	574(58)		118(59.9)		344(48.2)	
ER+/PR-	100(10.1)		18(9.1)		70(9.8)	
ER-/PR-	125(12.6)		19(9.6)		97(13.6)	
Unknown	191(19.3)		42(21.3)		202(28.3)	

Abbreviations: BMI = body mass index, PMD = percent mammographic density, V = variation measure, HT = Postmenopausal hormone therapy, ER = estrogen receptor, PR = progesterone receptor, NHS = Nurses' Health Study, NHSII = Nurses' Health Study II

Supplementary Table 2. Participant characteristics at time of mammogram by PMD, APD, and V among controls in the NHS/NHSII

	Manual percent MD				Automated percent MD				V			
	Quartile 1 (N = 981)	Quartile 2 (N = 979)	Quartile 3 (N = 980)	Quartile 4 (N = 981)	Quartile 1 (N = 980)	Quartile 2 (N = 980)	Quartile 3 (N = 980)	Quartile 4 (N = 981)	Quartile 1 (N = 980)	Quartile 2 (N = 980)	Quartile 3 (N = 980)	Quartile 4 (N = 981)
Mean (SD)												
Age (years)	57.5(8.6)	54.1(9.0)	50.5(7.8)	48.4(7.2)	56.3(9.1)	53.4(9.2)	51.1(8.2)	49.6(7.6)	55.2(9.1)	53.5(9.0)	51.4(8.6)	50.3(8.1)
BMI (kg/m2)	29.0(5.9)	26.6(5.1)	24.9(4.5)	23.0(3.5)	28.7(6.1)	26.5(5.5)	24.9(4.4)	23.5(3.3)	28.5(6.0)	26.5(5.6)	24.5(4.3)	24.1(3.8)
PMD	8.8(4.6)	23.0(3.8)	37.7(4.5)	59.0(9.6)	15.7(14.9)	27.4(17.4)	37.9(16.3)	47.5(13.3)	17.0(15.5)	27.9(17.8)	38.2(17.5)	45.5(14.4)
APD	12.7(3.8)	16.7(3.6)	19.8(3.2)	20.8(3.4)	10.9(2.4)	16.4(1.2)	19.8(0.8)	23(1.3)	11.6(3.1)	16.8(2.8)	19.9(2.5)	21.8(2.3)
Variation measure	-1.0(0.7)	-0.3(0.8)	0.3(0.8)	0.5(0.8)	-1.2(0.6)	-0.3(0.6)	0.3(0.6)	0.8(0.6)	-1.3(0.5)	-0.4(0.2)	0.2(0.2)	1.1(0.5)
Mammogram year	1994.7(4.6)	1996.0(5.0)	1996.6(4.8)	1996.1(4.5)	1994.9(4.9)	1996.2(5.1)	1996.2(4.7)	1996(4.4)	1995.1(4.8)	1995.5(4.7)	1995.8(4.7)	1996.9(4.7)
N (%)												
Cohort												
NHS	773(78.8)	590(60.2)	417(42.6)	383(39.0)	711(72.6)	544(55.5)	478(48.8)	430(43.8)	669(68.3)	566(57.8)	502(51.2)	426(43.4)
NHSII	208(21.1)	390(39.8)	563(57.4)	598(61.0)	269(27.4)	436(44.5)	502(51.2)	551(56.2)	311(31.7)	414(42.2)	478(48.8)	555(56.6)
Menopausal status												
Premenopausal	247(25.2)	378(38.6)	567(57.9)	685(69.8)	306(31.2)	428(43.7)	517(52.8)	626(63.8)	324(33.1)	415(42.3)	535(54.6)	603(61.5)
Postmenopausal	672(68.6)	535(54.6)	352(35.9)	245(25.0)	617(63.0)	490(50.0)	397(40.5)	300(30.6)	593(60.5)	504(51.4)	385(39.3)	322(32.8)
Unknown	61(6.2)	67(6.8)	61(6.2)	51(5.2)	57(5.8)	62(6.3)	66(6.7)	55(5.6)	63(6.4)	61(6.2)	60(6.1)	56(5.7)
HT use												
Never	482(49.2)	465(47.4)	533(54.4)	600(61.2)	494(50.4)	510(52.0)	518(52.9)	558(56.9)	513(52.3)	481(49.1)	536(54.7)	550(56.1)
Past	275(28.1)	295(30.1)	277(28.3)	221(22.5)	260(26.5)	264(26.9)	291(29.7)	253(25.8)	264(26.9)	268(27.3)	273(27.9)	263(26.8)
Current	192(19.6)	174(17.8)	129(13.2)	126(12.8)	184(18.8)	168(17.1)	133(13.6)	136(13.9)	163(16.6)	184(18.8)	142(14.5)	132(13.5)
Unknown	31(3.2)	46(4.7)	41(4.2)	34(3.5)	42(4.3)	38(3.9)	38(3.9)	34(3.5)	40(4.1)	47(4.8)	29(3.0)	36(3.7)

Abbreviations: BMI = body mass index, PMD = percent mammographic density, V = variation measure, HT = Postmenopausal hormone therapy, ER = estrogen receptor, PR = progesterone receptor, NHS = Nurses' Health Study, NHSII = Nurses' Health Study II

Supplementary Table 3. Odds ratios and 95% confidence intervals for the associations between APD, PMD, V, and breast cancer risk by ER/PR status, in the NHS/NHSII*

	ER+/PR+<br>N = 1036	ER+/PR-<br>N = 188	ER-/PR-<br>N = 241	p-het
PMD				
Model 1[1]				
Quartile 1<br><16	Ref	Ref	Ref	
Quartile 2<br>16 - <30	1.51<br>(1.21 to 1.88)	1.12<br>(0.71 to 1.79)	1.39<br>(0.91 to 2.14)	
Quartile 3<br>30 - <46	1.83<br>(1.45 to 2.31)	1.70<br>(1.06 to 2.73)	1.82<br>(1.17 to 2.83)	
Quartile 4<br>≥ 46	2.77<br>(2.18 to 3.53)	2.69<br>(1.66 to 4.37)	2.43<br>(1.54 to 3.84)	
p-trend	<0.01	<0.01	<0.01	
Per 1 SD	1.46<br>(1.34 to 1.59)	1.69<br>(1.41 to 2.01)	1.43<br>(1.22 to 1.67)	0.29
Model 1 + V				
Quartile 1<br><16	Ref	Ref	Ref	
Quartile 2<br>16 - <30	1.35<br>(1.08 to 1.70)	1.11<br>(0.69 to 1.80)	1.18<br>(0.76 to 1.85)	
Quartile 3<br>30 - <46	1.49<br>(1.16 to 1.93)	1.67<br>(0.99 to 2.81)	1.35<br>(0.83 to 2.20)	
Quartile 4<br>≥ 46	2.21<br>(1.68 to 2.89)	2.63<br>(1.52 to 4.55)	1.73<br>(1.03 to 2.90)	
p-trend	<0.01	<0.01	0.02	
Per 1 SD	1.36<br>(1.24 to 1.49)	1.68<br>(1.39 to 2.03)	1.31<br>(1.11 to 1.56)	0.09
APD				
Model 1[1]				
Quartile 1<br><14	Ref	Ref	Ref	
Quartile 2<br>14 - <18	1.55<br>(1.25 to 1.92)	1.42<br>(0.93 to 2.18)	1.49<br>(0.98 to 2.27)	
Quartile 3<br>18- <21	1.55<br>(1.23 to 1.94)	1.13<br>(0.71 to 1.81)	1.50<br>(0.97 to 2.32)	
Quartile 4<br>≥ 21	2.16<br>(1.72 to 2.70)	1.61<br>(1.01 to 2.55)	2.06<br>(1.34 to 3.16)	
p-trend	<0.01	0.12	<0.01	
Per 1 SD	1.33<br>(1.23 to 1.45)	1.21<br>(1.02 to 1.43)	1.36<br>(1.17 to 1.59)	0.53
Model 1 + V				
Quartile 1<br><14	Ref	Ref	Ref	
Quartile 2<br>14 - <18	1.30<br>(1.02 to 1.64)	1.23<br>(0.77 to 1.99)	1.13<br>(0.71 to 1.78)	
Quartile 3<br>18- <21	1.14<br>(0.86 to 1.51)	0.89<br>(0.49 to 1.61)	0.93<br>(0.54 to 1.59)	
Quartile 4<br>≥ 21	1.44<br>(1.05 to 1.98)	1.16<br>(0.59 to 2.28)	1.09<br>(0.60 to 1.99)	
p-trend	0.09	0.85	0.64	
Per 1 SD	1.16<br>(1.02 to 1.32)	1.12<br>(0.84 to 1.48)	1.17<br>(0.91 to 1.51)	0.96
V				
Model 1[1]				
Quartile 1<br>< -0.76	Ref	Ref	Ref	
Quartile 2<br>-0.76 - < -0.09	1.31<br>(1.05 to 1.62)	1.70<br>(1.09 to 2.66)	1.44<br>(0.94 to 2.21)	
Quartile 3<br>-0.09 – 0.56	1.63<br>(1.31 to 2.02)	1.59<br>(1.00 to 2.54)	1.62<br>(1.06 to 2.50)	
Quartile 4<br>> 0.56	2.09<br>(1.69 to 2.60)	1.77<br>(1.11 to 2.85)	2.24<br>(1.48 to 3.41)	
p-trend	<0.01	0.02	<0.01	
Per 1 SD	1.32<br>(1.22 to 1.42)	1.20<br>(1.02 to 1.42)	1.35<br>(1.17 to 1.56)	0.53
Model 1 + PMD				
Quartile 1<br>< -0.76	Ref	Ref	Ref	
Quartile 2<br>-0.76 - < -0.09	1.17<br>(0.94 to 1.45)	1.41<br>(0.90 to 2.22)	1.29<br>(0.84 to 1.99)	
Quartile 3<br>-0.09 – 0.56	1.31<br>(1.04 to 1.65)	1.12<br>(0.69 to 1.82)	1.32<br>(0.84 to 2.06)	
Quartile 4<br>> 0.56	1.55<br>(1.22 to 1.96)	1.07<br>(0.65 to 1.78)	1.68<br>(1.07 to 2.63)	
p-trend	<0.01	0.97	0.04	

Per 1 SD	1.18<br>(1.08 to 1.28)	1.00<br>(0.84 to 1.19)	1.22<br>(1.04 to 1.42)	0.17
Model 1 + APD				
Quartile 1<br>< -0.76	Ref	Ref	Ref	
Quartile 2<br>-0.76 - < -0.09	1.10<br>(0.85 to 1.41)	1.52<br>(0.90 to 2.58)	1.20<br>(0.73 to 1.97)	
Quartile 3<br>-0.09 – 0.56	1.24<br>(0.92 to 1.67)	1.34<br>(0.71 to 2.54)	1.22<br>(0.68 to 2.19)	
Quartile 4<br>> 0.56	1.49<br>(1.07 to 2.08)	1.43<br>(0.70 to 2.95)	1.58<br>(0.83 to 3.01)	
p-trend	0.01	0.40	0.37	
Per 1 SD	1.18<br>(1.04 to 1.34)	1.11<br>(0.84 to 1.45)	1.20<br>(0.95 to 1.51)	0.89

Abbreviations: BMI = body mass index; PMD = percent mammographic density; V = variation measure; NHS = Nurses' Health Study; NHSII = Nurses' Health Study II; ER = estrogen receptor; PR = progesterone receptor.

1. Models are adjusted for: Age (continuous), BMI (continuous), fasting status, time of blood draw, menopausal status (premenopausal, postmenopausal, unknown), hormone therapy use (never, past, current, unknown), mammography read batch (batch 1, batch 2, batch 3)

Supplementary Table 4. Odds ratios and 95% confidence intervals for the associations between PMD, APD, V, and breast cancer risk according to menopausal status in the NHS/NHSII, high resolution images only[1]

PMD						
	Premenopausal			Postmenopausal		
	Model 1	*Model 1 + V*		*Model 1*	*Model 1 + V*	
Quartile 1<br><16	Ref	Ref		Ref	Ref	
Quartile 2<br>16 - <30	2.18<br>(1.24 to 3.83)	1.90<br>(1.08 to 3.37)		1.40<br>(0.97 to 2.02)	1.16<br>(0.79 to 1.70)	
Quartile 3<br>30 - <46	2.90<br>(1.68 to 4.99)	2.22<br>(1.26 to 3.94)		1.82<br>(1.22 to 2.72)	1.34<br>(0.86 to 2.09)	
Quartile 4<br>≥ 46	5.07<br>(2.93 to 8.78)	3.82<br>(2.14 to 6.82)		2.24<br>(1.43 to 3.53)	1.58<br>(0.96 to 2.61)	
p-trend	<0.01	<0.01		<0.01	0.06	
Per 1 SD	1.63<br>(1.43 to 1.85)	1.53<br>(1.34 to 1.75)		1.46<br>(1.21 to 1.75)	1.28<br>(1.05 to 1.56)	
APD						
	Premenopausal			Postmenopausal		
	Model 1	*Model 1 + V*		*Model 1*	*Model 1 + V*	
Quartile 1<br><14	Ref	Ref		Ref	Ref	
Quartile 2<br>14 - <18	1.93<br>(1.23 to 3.03)	1.65<br>(1.03 to 2.64)		1.65<br>(1.11 to 2.44)	1.21<br>(0.78 to 1.87)	
Quartile 3<br>18- <21	2.76<br>(1.80 to 4.25)	2.09<br>(1.28 to 3.42)		1.13<br>(0.74 to 1.73)	0.65<br>(0.38 to 1.12)	
Quartile 4<br>≥ 21	3.04<br>(1.98 to 4.66)	2.10<br>(1.23 to 3.58)		2.40<br>(1.57 to 3.65)	1.13<br>(0.61 to 2.10)	
p-trend	<0.01	0.01		<0.01	0.86	
Per 1 SD	1.49<br>(1.30 to 1.71)	1.31<br>(1.08 to 1.58)		1.35<br>(1.16 to 1.58)	1.05<br>(0.82 to 1.35)	
V						
	Premenopausal			Postmenopausal		
	Model 1	*Model 1 + PMD*	*Model 1 + APD*	*Model 1*	*Model 1 + PMD*	*Model 1 + APD*
Quartile 1<br>< -0.76	Ref	Ref	Ref	Ref	Ref	Ref
Quartile 2<br>-0.76 - <-0.09	1.98<br>(1.34 to 2.91)	1.68<br>(1.14 to 2.49)	1.49<br>(0.96 to 2.31)	1.01<br>(0.70 to 1.47)	0.93<br>(0.63 to 1.35)	0.83<br>(0.53 to 1.32)
Quartile 3<br>-0.09 – 0.56	2.12<br>(1.46 to 3.09)	1.64<br>(1.11 to 2.41)	1.37<br>(0.84 to 2.24)	1.59<br>(1.08 to 2.33)	1.36<br>(0.91 to 2.03)	1.20<br>(0.70 to 2.06)
Quartile 4<br>> 0.56	2.78<br>(1.91 to 4.03)	2.08<br>(1.42 to 3.05)	1.67<br>(0.99 to 2.81)	2.23<br>(1.50 to 3.33)	1.80<br>(1.18 to 2.77)	1.60<br>(0.87 to 2.92)
p-trend	<0.01	<0.01	0.10	<0.01	<0.01	0.03
Per 1 SD	1.37<br>(1.22 to 1.53)	1.25<br>(1.11 to 1.40)	1.17<br>(1.00 to 1.37)	1.41<br>(1.22 to 1.64)	1.30<br>(1.11 to 1.53)	1.36<br>(1.07 to 1.72)

Abbreviations: BMI = body mass index; PMD = percent mammographic density; V = variation measure; NHS = Nurses' Health Study; NHSII = Nurses' Health Study II

1. Models are adjusted for: Age (continuous), BMI (continuous), fasting status, time of blood draw, hormone therapy use (never, past, current, unknown—postmenopausal models only), mammography read batch (batch 1, batch 2, batch 3)

Supplementary Table 5. Odds ratios and 95% confidence intervals for the associations between breast cancer risk and cross-classified PMD or APD and V, in the NHS/NHSII, high resolution images only1

	V				
PMD	Quartile 1<br>< -0.76	Quartile 2<br>-0.76 - < -0.09	Quartile 3<br>-0.09 – 0.56	Quartile 4<br>> 0.56	p-interaction
Quartile 1<br><16	Ref	1.78<br>(1.06 to 3.00)	1.60<br>(0.68 to 3.80)	3.16 (0.51 to 19.6)	0.51
Quartile 2<br>16 - <30	1.56<br>(0.98 to 2.49)	1.81<br>(1.18 to 2.78)	2.71<br>(1.73 to 4.24)	2.53<br>(1.48 to 4.30)	
Quartile 3<br>30 - <46	2.37<br>(1.31 to 4.29)	2.15<br>(1.36 to 3.40)	2.24<br>(1.47 to 3.43)	3.48<br>(2.31 to 5.25)	
Quartile 4<br>≥ 46	3.68<br>(1.88 to 7.20)	3.99<br>(2.49 to 6.38)	4.17<br>(2.74 to 6.35)	4.94<br>(3.30 to 7.40)	
APD					
Quartile 1<br><14	Ref	1.25<br>(0.61 to 2.55)	2.20<br>(0.55 to 8.80)	2.57<br>(0.23 to 29.1)	0.53
Quartile 2<br>14 - <18	1.91 (1.28 to 2.86)	1.80<br>(1.27 to 2.55)	2.31<br>(1.46 to 3.68)	2.47<br>(1.27 to 4.79)	
Quartile 3<br>18- <21	0.81<br>(0.18 to 3.71)	1.81<br>(1.25 to 2.63)	2.00<br>(1.41 to 2.85)	2.86<br>(1.97 to 4.16)	
Quartile 4<br>≥ 21	Not estimated	2.46<br>(1.44 to 4.23)	2.48<br>(1.75 to 3.52)	3.06<br>(2.21 to 4.22)	

Abbreviations: BMI = body mass index; PMD = percent mammographic density; V = variation measure; NHS = Nurses' Health Study; NHSII = Nurses' Health Study II

1. Models are adjusted for: Age (continuous), BMI (continuous), fasting status, time of blood draw, menopausal status (premenopausal, postmenopausal, unknown), hormone therapy use (never, past, current, unknown), mammography read batch (batch 1, batch 2, batch 3)
