# Supplementary material for: Automated percent mammographic density, mammographic texture variation, and risk of breast cancer: a nested case-control study
Source: NPJ Breast Cancer. 2021 May 31;7:68. doi: 10.1038/s41523-021-00272-2 (PMC8166859; doi:10.1038/s41523-021-00272-2)
Supplement: Supplementary file 2 — Reporting Summary [file 41523_2021_272_MOESM2_ESM.pdf]

nature research

Corresponding author(s): Erica T.Warner

Last updated by author(s): Oct 8, 2020

Reporting Summary

Nature Research wishes to improve the reproducibility of the work that we publish. This form provides structure for consistency and transparency in reporting. For further information on Nature Research policies, see our Editorial Policies and the Editorial Policy Checklist.

Statistics

For all statistical analyses, confirm that the following items are present in the figure legend, table legend, main text, or Methods section.

n/a	Confirmed	
☐	☒	The exact sample size (n) for each experimental group/condition, given as a discrete number and unit of measurement
☒	☐	A statement on whether measurements were taken from distinct samples or whether the same sample was measured repeatedly
☐	☒	The statistical test(s) used AND whether they are one- or two-sided<br>*Only common tests should be described solely by name; describe more complex techniques in the Methods section.*
☐	☒	A description of all covariates tested
☐	☒	A description of any assumptions or corrections, such as tests of normality and adjustment for multiple comparisons
☐	☒	A full description of the statistical parameters including central tendency (e.g. means) or other basic estimates (e.g. regression coefficient) AND variation (e.g. standard deviation) or associated estimates of uncertainty (e.g. confidence intervals)
☐	☒	For null hypothesis testing, the test statistic (e.g. F, t, r) with confidence intervals, effect sizes, degrees of freedom and P value noted<br>*Give P values as exact values whenever suitable.*
☒	☐	For Bayesian analysis, information on the choice of priors and Markov chain Monte Carlo settings
☒	☐	For hierarchical and complex designs, identification of the appropriate level for tests and full reporting of outcomes
☐	☒	Estimates of effect sizes (e.g. Cohen's d, Pearson's r), indicating how they were calculated

Our web collection on statistics for biologists contains articles on many of the points above.

Software and code

Policy information about availability of computer code

Data collection	Algorithms used to generate automated percent density and V are available upon request to the corresponding author.
Data analysis	All data analysis performed using SAS version 9.4 as described in Methods.

For manuscripts utilizing custom algorithms or software that are central to the research but not yet described in published literature, software must be made available to editors and reviewers. We strongly encourage code deposition in a community repository (e.g. GitHub). See the Nature Research guidelines for submitting code & software for further information.

Data

Policy information about availability of data

All manuscripts must include a data availability statement. This statement should provide the following information, where applicable:

- Accession codes, unique identifiers, or web links for publicly available datasets
- A list of figures that have associated raw data
- A description of any restrictions on data availability

The data that support the findings of this study are available from the Nurses' Health Studies but restrictions apply to the availability of these data, and so they are not publicly available. However, data are available from the authors upon reasonable request and with permission of Nurses' Health Studies External Advisory Board. Additional data sharing information and policy details can be accessed at http://www.nurseshealthstudy.org/researchers.

Field-specific reporting

Please select the one below that is the best fit for your research. If you are not sure, read the appropriate sections before making your selection.

☒ Life sciences ☐ Behavioural & social sciences ☐ Ecological, evolutionary & environmental sciences

For a reference copy of the document with all sections, see nature.com/documents/nr-reporting-summary-flat.pdf

Life sciences study design

All studies must disclose on these points even when the disclosure is negative.

Sample size	The sample size was based on Nurses Health Study and Nurses Health Study II participants in the nested case control studyN/A with available mammograms
Data exclusions	Mammograms were collected from 2062 breast cancer cases and 4196 matched controls. We excluded 162 cases and 275 controls due to missing data on V or BMI.
Replication	N/A
Randomization	N/A this was an observational study
Blinding	N/A this was an observational study

Reporting for specific materials, systems and methods

We require information from authors about some types of materials, experimental systems and methods used in many studies. Here, indicate whether each material, system or method listed is relevant to your study. If you are not sure if a list item applies to your research, read the appropriate section before selecting a response.

Materials & experimental systems

n/a	Involved in the study	
☒	☐	Antibodies
☒	☐	Eukaryotic cell lines
☒	☐	Palaeontology and archaeology
☒	☐	Animals and other organisms
☐	☒	Human research participants
☒	☐	Clinical data
☒	☐	Dual use research of concern

Methods

n/a	Involved in the study	
☒	☐	ChIP-seq
☒	☐	Flow cytometry
☒	☐	MRI-based neuroimaging

Human research participants

Policy information about studies involving human research participants

Population characteristics	Our study population includes 1900 breast cancer cases and 3921 matched controls from the Nurses' Health Study (NHS) and Nurses' Health Study II (NHSII). NHS began in 1976 with 121,700 female registered nurses aged 30 to 55 from 11 states. NHSII began in 1989 with 116,429 female registered nurses, aged 25 to 42, from 14 states. Participants in each cohort completed a baseline questionnaire at enrollment and are followed by biennially mailed questionnaires to collect information on newly diagnosed diseases, exposures, and covariates.
Recruitment	Female registered nurses were recruited through nursing associations and registries
Ethics oversight	The study protocol was approved by the institutional review boards of the Brigham and Women's Hospital and Harvard T.H. Chan School of Public Health, and those of participating registries as required.

Note that full information on the approval of the study protocol must also be provided in the manuscript.
